# Synthesis, Properties and Stereochemistry of 2-Halo-1,2λ^5^-oxaphosphetanes

**DOI:** 10.3390/molecules21101371

**Published:** 2016-10-17

**Authors:** Anastasy O. Kolodiazhna, Oleg I. Kolodiazhnyi

**Affiliations:** Institute of Bioorganic Chemistry and Petrochemistry, National Academy of Sciences of Ukraine, 1, Murmanska Street, Kiev 02094, Ukraine; anastasik7@rambler.ru

**Keywords:** 2-halo-1,2λ^5^-oxaphosphetanes, allylphosphonates, vinylphosphonates, phosphorus ketenes

## Abstract

Results of research into four-membered 2-halo-1,2λ^5^-oxaphosphetane phosphorus(V)-heterocycles are presented. The preparation of 2-halo-1,2λ^5^-oxaphosphetanes by reaction of *P-*haloylides with carbonyl compounds is described. The mechanism of asynchronous [2+2]-сycloaddition of ylides to aldehydes was proposed on the base of low-temperature NMR investigations. 2-Halo-1,2λ^5^-oxaphosphetanes were isolated as individual compounds and their structures were confirmed by ^1^Н-, ^13^C-, ^19^F- and ^31^Р-NMR spectra. These compounds are convenient reagents for preparing of various organic and organophosphorus compounds hardly available by other methods. Chemical and physical properties of the 2-halo-1,2λ^5^-oxaphosphetanes are reviewed. The 2-chloro-1,2λ^5^-oxaphosphetanes, rearrange with formation of 2-chloroalkyl-phosphonates or convert into *trans*-phosphorylated alkenes depending on the substituents at the α-carbon atom. Prospective synthetic applications of 2-halo-1,2λ^5^-oxaphosphetanes are analyzed. The 2-halo-1,2λ^5^-oxaphosphetanes may be easily converted to various alkenylphosphonates: allyl- or vinylphosphonates, phosphorus ketenes, thioketenes, ketenimines.

## 1. Introduction

One of the most interesting and intriguing classes of organophosphorus compounds are the 1,2-oxaphosphetanes—four-membered heterocycles containing pentacoordinated phosphorus [1,2,3,4,5,6,7]. Since 1,2-oxaphosphetanes are well-known intermediates in the Wittig reaction, a number of efforts have been made for their structural characterization both in solution and the solid state [8,9,10,11,12,13].

**Scheme 1:**
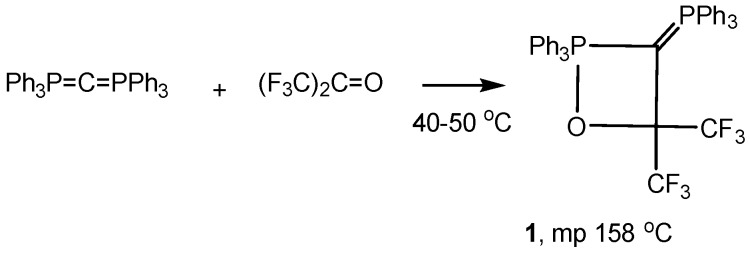
First representative of stable 1,2-oxaphosphetane**s** isolated by Birum and Matthews.

In 1967, Birum and Matthews had already reported the structural characterization (NMR and X-ray study) of the first isolated 1,2-oxaphosphetane **1**. Compound **1** was prepared in 76% yield by allowing hexaphenylcarbodiphosphorane to react with hexafluoroacetone in dry diglyme [14] (Scheme 1).

**Scheme 2:**
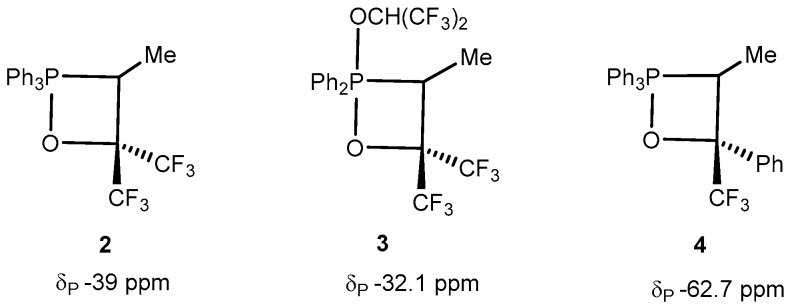
1,2-oxaphosphetanes **2**–**4** registered by low temperature NMR spectroscopy.

Vedejs [3,4] succeeded in detecting of 1,2-oxaphosphetanes **2**–**4** by low temperature NMR spectroscopy during typical Wittig reactions and observed that these intermediates readily decompose upon warming to room temperature into alkenes and phosphine oxides (Scheme 2).

**Scheme 3:**
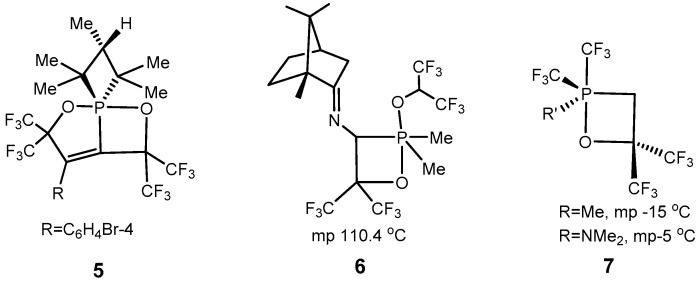
Stabilized bis(trifluoromethylated) oxaphosphetanes **5**,**6**.

Schmutzler and co-workers reported several stabilized bis(trifluoromethylated) oxaphosphetanes **5**, **6** (Scheme 3) [15] which were characterized by NMR, MS spectra and X-ray analysis. At room temperature Berry-pseudorotation was fast on the NMR time scale, impeding one from distinguishing apical and equatorial P-CF_3_ groups. Decreasing the temperature to −60 °C in toluene-*d*_8_ allowed resolving the signals for all CF_3_ groups of molecule **7** [16].

**Scheme 4:**
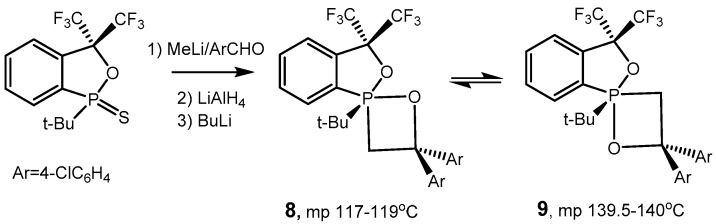
Stereomutation of anti-apicophilic oxaphosphetane **8**.

Kojima [17] reported the interesting anti-apicophilic spirophosphorane **8** bearing an oxaphosphetane ring. The structure of compounds **8** was confirmed by X-ray diffraction. Crystallization from hexane gave the pure anti-apicophilic derivative. Stereomutation of compound **8** was observed in the presence of acids and slowed down when DBU was present, suggesting that the isomerization into **9** is rather the result of a P–O bond breaking-recombination process. Evidently, this conversion represents an example of a thermodynamically stable oxaphosphetanes, in which pseudorotation is faster than alkene formation (Scheme 4).

**Scheme 5:**
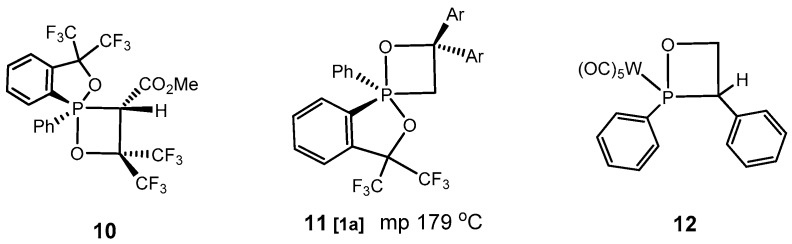
Oxaphosphetanes stabilized by CF_3_ groups **10**,**11** and 1,2-oxaphosphetane complex **12**.

Most of the previously reported stable oxaphosphetane structures contain fluorine-bearing or bicyclic phosphole-type ligands either at the phosphorus position or at the 4 position in the oxaphosphetane ring **10**, **11** [15,16,17,18,19,20]. Recently, Streubel and coworkers have prepared the first 1,2-oxaphosphetane complexes **12** formally similar to traditional oxaphosphetanes, using low-temperature ring expansion of epoxides with a Li/Cl phosphinidenoid complex [18] (Scheme 5).

**Scheme 6:**
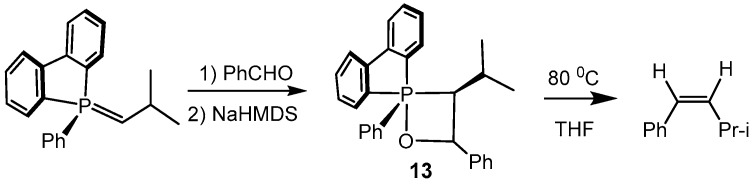
Stereospecific decomposition of oxaphosphetane **13**.

Gilheany studied oxaphosphetane intermediates in the Wittig reaction by variable-temperature NMR spectroscopy [9,10,11]. Compound **13** was obtained by low-temperature acid quenching of the Wittig reaction of ylide with benzaldehyde, a suitable representative aromatic aldehyde (see Scheme 6). The major diastereomer was the *syn*-**13** on the basis that the unquenched Wittig reaction gives the (*Z*)-alkene as the major product. In this manner, the *syn/anti* ratio of **13** was 89:11.

Keglevich reported detection of enantiomers of *P-*stereogenic pentacoordinated phosphorus compounds [20]. Detailed ^31^P-NMR investigations of oxaphosphetes in optically active solvents have clearly shown that the most electronegative substituents (e.g., oxygen) prefers the apical position in a trigonal bipyramidal structure and that the pentacoordinated phosphorus atom is in a dynamic condition due to pseudorotation. Berger and coworkers found that 2-furyl groups on the phosphorus atom increase the thermal stabilities of oxaphosphetanes and succeeded in isolation and determination of the X-ray structure of tris(2-furyl) substituted oxaphosphetane, the stability of which is attributed to the electron-withdrawing properties of the 2-furyl group [21].

**Scheme 7:**
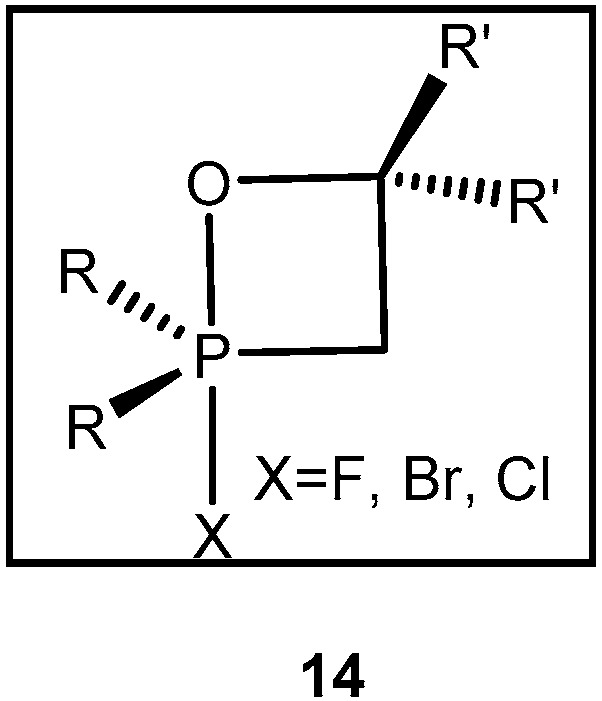
Examples of stable oxaphosphetanes.

Among the stable oxaphosphetanes [1,2,3,4,5,6,7,8,9,10,11,12,13,14,15,16,17,18,19,20,21,22], 2-halo-1,2λ^5^-oxaphosphetanes **14**, which possess relatively high stability and diverse reactivity, attract particular interest [23,24,25,26,27,28,29,30,31,32,33,34,35,36,37,38] (Scheme 7). These oxaphosphetanes containing fluorine, chlorine or bromine atoms bonded to phosphorus are an interesting class of pentacoordinated phosphorus heterocycles possessing peculiar properties. The chemical properties of 2-halo-1,2λ^5^-oxaphosphetanes, first of all of *P-*chloro- and *P-*fluoroylides, due to the presence of a labile halogen atom on phosphorus, are very specific and differ from the properties of triphenylphosphonium ylides. For example, reactions and conversions of 2-halo-1,2λ^5^-oxaphosphetanes proceed with pres**e**rvation of the P-C bond and leads to the formation of different organophosphorus compounds. In addition, this type of compounds exhibit also physico-chemical properties uncharacteristic for traditional *P,P,P*-triorganosubstituted oxaphosphetanes. For the first time the 2-halo-1,2λ^5^-oxaphosphetanes were prepared in our laboratory almost twenty years ago and up to today we and some other authors are still studying their chemistry. In this article, we summarize the synthesis and properties of this type of organophosphorus compounds. The chemistry of 2-halo-1,2λ^5^-oxaphosphetanes was not previously analyzed, generalized or reviewed.

## 2. Results and Discussion

### 2.1. General Description

Since pentacoordinated phosphoranes formally have 10 electrons in the valence shell, they display a specific bonding model. Therefore pentacoordinated phosphoranes take a trigonal bipyramidal structure and there are two ligating sites, apical and equatorial sites. The apical bond consists of three-center four-electron bond using the *p* orbital of the central phosphorus atom, while the equatorial bond is a typical *s* bond using *sp*^2^ hybrid orbital of the phosphorus atom. This three-center four-electron bond forms three molecular orbitals.

It is generally known that pentacoordinated phosphoranes rapidly undergo intramolecular positional isomerization without bond cleavage. A very rapid non-dissociative intramolecular site exchange is usually explained by the Berry pseudorotation mechanism [19,22,23].

The 2-halo-1,2λ^5^-oxaphosphetanes (halogen = chlorine, bromine, fluorine) are the most stable representatives of this type of compounds. They can be purified by distillation under vacuum and stored in a refrigerator. At the same time they possess interesting chemical properties and participate in various chemical transformations [24,25,26,27,28].

The stability of 2-halo-oxaphosphetanes changes in the same sequence of substituents R^4^ at the endocyclic carbon atoms at position 4. The most stable are compounds containing strong electron-accepting groups at C(4), drawing off electron density from the oxygen atom as a result of which the three-centre apical bond O—P—Hal is strengthened. Chloro-oxaphosphetanes, having less electron-accepting substituents at C(4), are dissociated to a large extent and correspondingly are converted into vinylphosphine oxides at room temperature.

### 2.2. Synthesis of 2-Halo-1,2λ^5^-oxaphosphetane

**Scheme 8:**
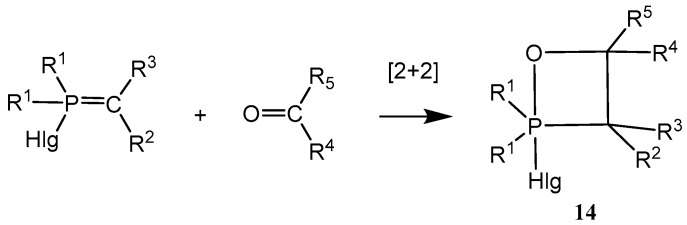
Synthesis of 2-halo-1,2λ^5^-oxaphosphetanes. Hlg = F, Cl, Br; R^1^ = Alk, Ph; CR^2^R^3^ = CH_2_, CHAlk, CAlk_2_, CHPh, CHSiMe_3_, CCl_2_, CBr_2_; CR^4^R^5^ = C=O, CNPh, CHAlk, CAlk_2_, CHPh, CPh_2_, CH_2_CH = CH_2_.

Available methods for the synthesis of 2-halo-1,2λ^5^-oxaphosphetanes can be used for investigation of the reaction mechanism of phosphorus ylides with carbonyl compounds as well as for preparing stable oxaphosphetanes that can be used as reactants for organic synthesis. The 2-halo-1,2λ^5^-oxaphosphetanes were prepared by reaction of *P-*fluoro-, chloro- or bromoylides with carbonyl compounds. *P-*Chloro- and *P-*bromoylides react with active ketones, containing a trifluoromethyl group, with the formation of stable [2+2]-cycloaddition products, 2-chloro- or 2-bromo-1,2λ^5^-oxaphosphetenes **14** were isolated in yields close to quantitative as crystalline substances or as liquids distillable in vacuum (Scheme 8, Table 1) [29,30,31,32,33,34,35,36,37,38,39,40,41,42].

The addition of *P-*halogen-ylides to ketones proceeded stereoselectively and led predominantly to the formation of one of the possible 2-halo-1,2λ^5^-oxaphosphetane diastereomers. 2-Halo-1,2λ^5^-oxaphosphetanes dissociate at the *P-*halogen bond in solution with the formation of cyclic phosphonium salts, as a result of which an equilibrium is established between the forms with five- and four-coordinate phosphorus atoms. Dissociation of 2-halo-1,2λ^5^-oxaphosphetanes is enhanced by reducing the electron-accepting properties of substituents and also by increasing priority solvent. The 2-halo-1,2λ^5^-oxaphosphetanes containing electron-accepting groups at C(4) are distinctly stabler than oxaphosphetanes with alkyl groups in this position [25,26,27].

**Scheme 9:**
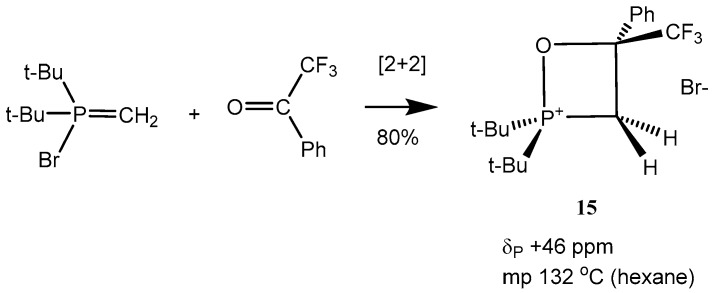
Ionized form of 2-bromo-1,2λ^5^-oxaphosphetanes **15**.

The reaction of *P-*bromomethylides with fluorinated acetophenone afforded in high yield oxaphosphetanes **15**, which were isolated as crystalline compounds (Scheme 9 and Table 1, entries 10–13). The compounds **15** exist in solution as cyclic phosphonium salt.

**Scheme 10:**
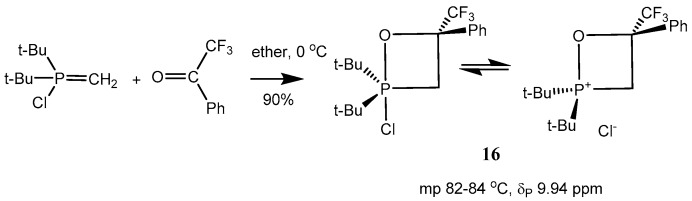
P(IV) and P(V)-forms of 2-chloro-1,2λ^5^-oxaphosphetane **16**.

**Scheme 11:**
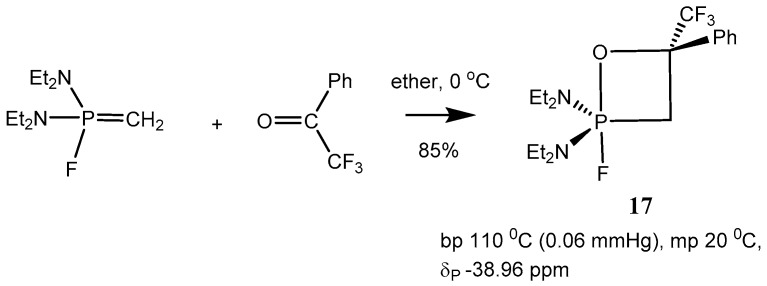
2-Fluoro-1,2λ^5^-oxaphosphetane **17**.

At the same time the 2-chloro-1,2λ^5^-oxaphosphetanes **16** exist as mixture of P(IV) and P(V)-forms. These compounds can be distilled under vacuum and dissolved in non-polar solvents (benzene) (Scheme 10 and Table 1, entries 1–9). Reaction of *P-*Fluoroylids with aldehydes and ketones proceeds in ether or pentane at −40–−20 °С and leads to the formation of stable 2-fluoro-1,2λ^5^-oxaphosphetanes **17** (Scheme 11 and Table 2).

The compounds **17** are liquids distilling in vacuo, the structure of which was proved by means of mass and NMR spectra. The ^31^Р-NMR spectra of 2-fluoro-oxaphosphetanes **17** present doublets with 800 Hz ^1^*J*_РF_ constants in the high magnetic field of a NMR spectrum at −37–−8 ppm. This corresponds to a pentacoordinate state of compounds **17** [24,26,27,28,29,30,31]. Tetracoordinated forms of 2-fluoro-oxaphosphetanes **17** were not registered by ^19^F- and ^31^P-NMR spectroscopy (Scheme 11).

**Scheme 12:**
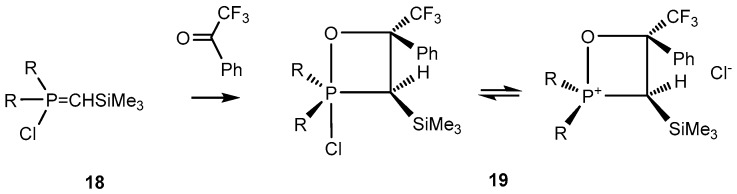
Silylated 2-halogene-1,2λ^5^-oxaphosphetanes **19**.

C-Silyl-*P-*chloroylides **18** react with carbonyl compounds to afford 2-chloro-1,2λ^5^-oxaphosphetanes **19**. The oxaphosphetanes **19** bearing an electronegative CF_3_ substituent at С-4 are relatively stable and can be isolated and analyzed by NMR (Scheme 12). The NMR spectra of these compounds reveal signals at 0.2 ppm, singlet (Me_3_Si), at 2 ppm, doublet, ^3^*J*_PH_ 18.0 Hz (С^3^Н), and at 4.5 ppm (С^4^Н). ^13^С-NMR signals of С-3 and С-4 carbons were found at 30 and 90 ppm, correspondingly. The ^31^P-NMR signals of 2-chloro-1,2λ^5^-oxaphosphetanes **19** at δ_P_ +48 ppm (R^1^ = *i*-PrO) and at δ_P_ = 60 ppm (R^1^ = Et_2_N) correspond to tetracoordinate phosphorus included in four-membered phosphetane cycle (Table 1) [25,32].

**Scheme 13:**
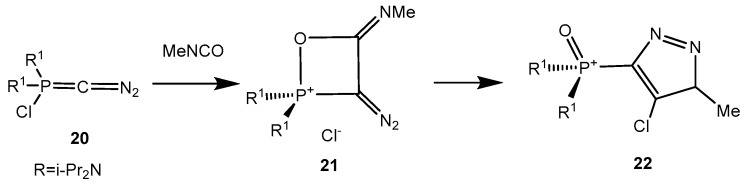
Adduct of phosphacumulene-ylide **20** with isocyanate.

**Scheme 14:**
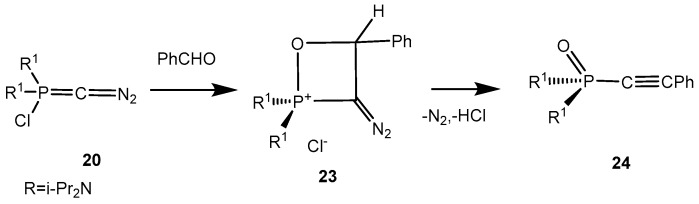
Reaction of phosphacumulene-ylide **20** with benzaldehyde.

Sotiropulos and Bertrand [33] reported the addition of phosphacumulene ylides **20** bearing the diazo group to isocyanates, leading to the formation of products **22**. It was proposed that initial nucleophilic attack of the ylide carbon atom on the carbonyl carbon gives a oxaphosphetane **21**, which depending on the relationship of the oxygen atom (or NR group) to nitrogen or phosphorus rearranges into products of 1,4- or 1,5-cyclisation **22** (Scheme 13). Benzaldehyde gives the 2-chlorooxaphosphetane **23** with the ylide **20** which readily eliminates hydrogen chloride and a nitrogen molecule being converted into an acetylene phosphonate **24** (Scheme 14).

**Scheme 15:**
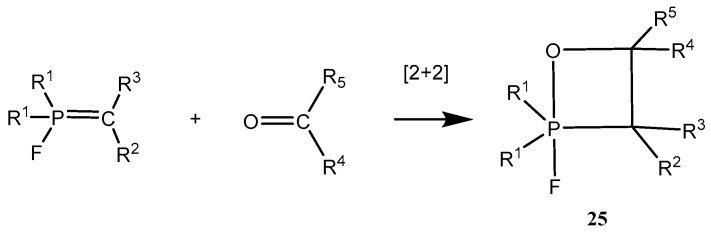
Synthesis of 2-fluoro-1,2λ^5^-oxaphosphetanes **25**. R^1^ = Alk, Ph; CR^2^R^3^ = CH_2_, CHAlk, CAlk_2_, CHPh, CHSiMe_3_, CCl_2_, CBr_2_; CR^4^R^5^ = C=O, CNPh, CHAlk, CAlk_2_, CHPh, CPh_2_, CH_2_CH = CH_2_.

A number of 2-fluoro-1,2λ^5^-oxaphosphetanes **25** were prepared by reaction of *P-*fluoroylides with aldehydes and ketones (Scheme 15, Table 2). The 2-fluoro-1,2λ^5^-oxaphosphetanes **25** are stable compounds, which can be isolated and purified by distillation under vacuum or by crystallization from non-polar solvents. These compounds are much distinguished from unstable adducts of carbonyl compounds with triphenylphosphonium ylides. Cycloadducts of *P-*fluoroylides with carbonyl compounds, 2-fluoro-1,2λ^5^-oxaphosphetanes, are also much more stable than 2-chloro- or 2-bromo-1,2λ^5^-oxaphosphetanes [25,26,27,28,29,30,31].

**Scheme 16:**
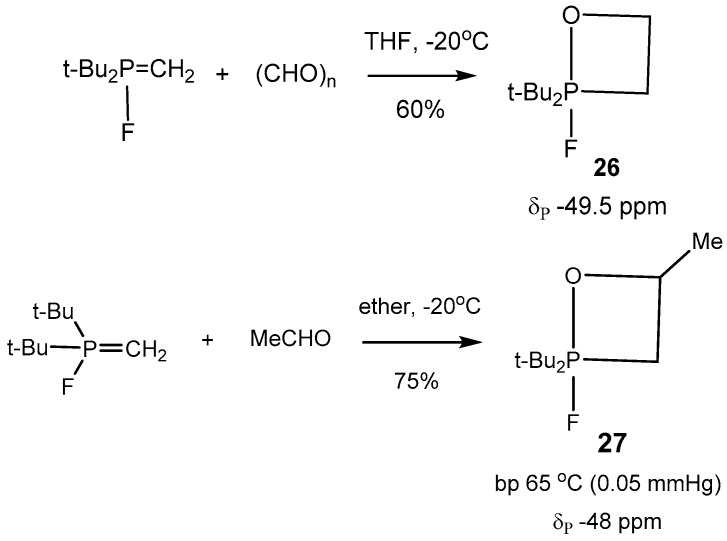
The simplest representatives of 2-Fluoro-1,2λ^5^-oxaphosphetanes **26**,**27**.

**Scheme 17:**
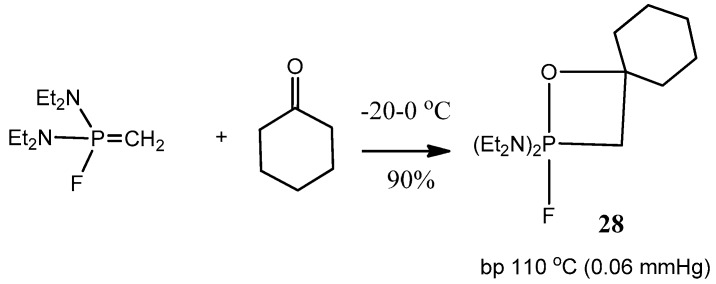
2-Fluoro-1,2λ^5^-oxaphosphetane **28**.

**Scheme 18:**
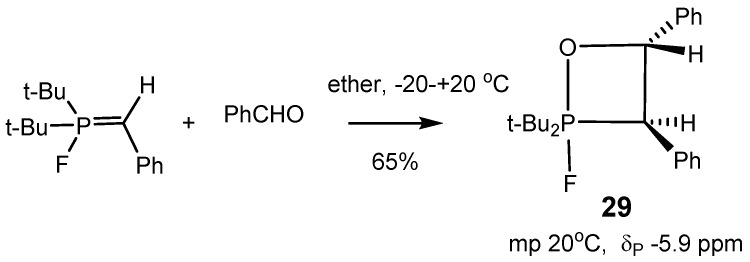
2-Fluoro-1,2λ^5^-oxaphosphetane **29**.

The stability of 2-fluoro-oxaphosphetanes is explained by the high electronegativity of the fluorine atom, compared to the electronegativities of chlorine and bromine. The P-F bond in 2-fluoro-1,2λ^5^-oxaphosphetanes is very strong, and, therefore, these compounds do not dissociate with formation of cyclic phosphonium salts, what is observed, for example, with 2-chloro-1,2λ^5^-oxaphosphetanes. Various stable 2-fluoro-1,2λ^5^-oxaphosphetanes were synthesized, isolated as pure specimens, and characterized (Table 2). Typical representatives of such compounds **26**–**29** are shown in Scheme 16, Scheme 17 and Scheme 18 [26,29].

The ^31^P-NMR spectra of compounds **25** show a doublet at −6 to −45 ppm with 760–850 Hz PF coupling constants appropriate for axial fluorine atoms. The ^31^P-NMR spectra of 2-fluoro-1,2λ^5^-oxaphosphetanes exhibit a doublet in the range −6 to −45 ppm, belonging to the five-coordinate phosphorus with corresponding coupling constants on axial fluorine atoms (760–850 Hz). The ^19^F-NMR spectra of 2-fluoro-1,2λ^5^-oxaphosphetanes exhibit doublets with the same PF coupling constants.

**Scheme 19:**
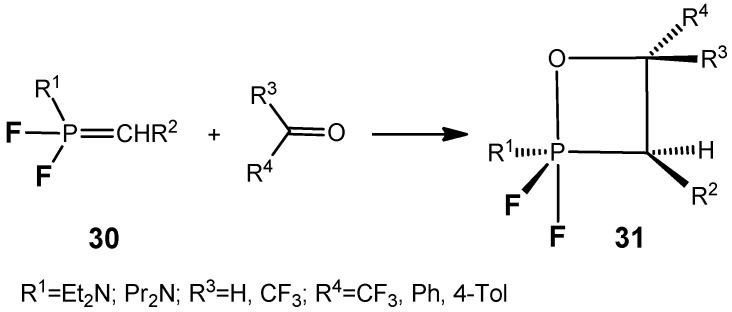
2,2-Difluoro-1,2λ^5^-oxaphosphetanes **31**.

The *P,P*-difluoroylides **30** react with aldehydes and active ketones to afford the stable oxaphosphetanes **31** bearing two fluorine atoms at the phosphorus (Scheme 19) [34,35,36]. The compounds **31** (R=CF_3_) were distilled under reduced pressure without decomposition and were characterized by means of NMR spectroscopy.

**Figure 1:**
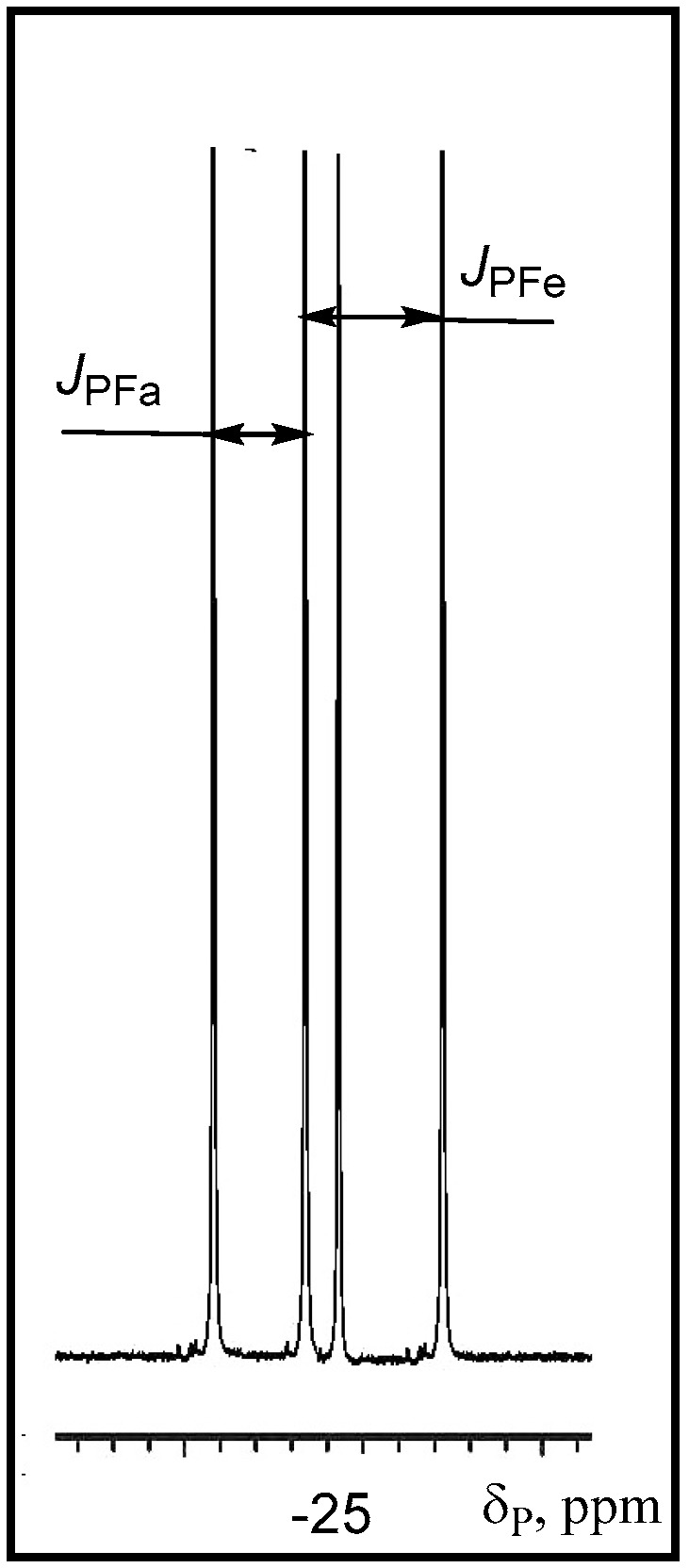
^31^P-NMR spectrum of 2-(diethylamino)-2,2-difluoro-4-tolyl-3-propyl-4-(trifluorophenyl-1,2λ^5^-oxaphosphetane **31** (See Table 2, entry 15).

**Figure 2:**
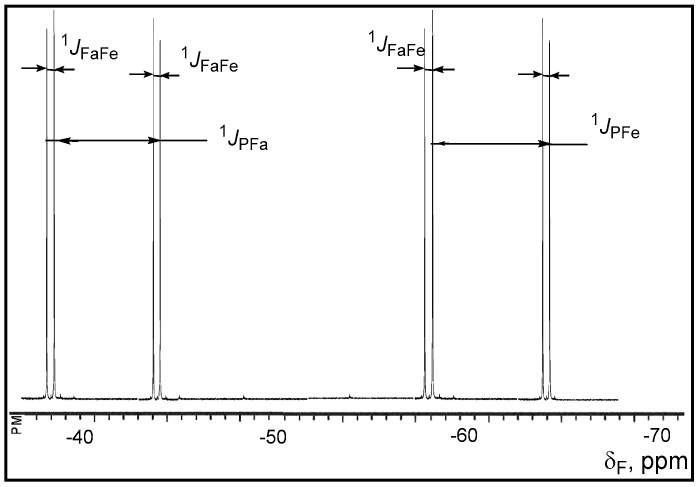
^19^F-NMR spectra of 2-(diethylamino)-2,2-difluoro-4-tolyl-3-propyl-4-(trifluorophenyl-1,2λ^5^-oxaphosphetane **31** (see Table 2, entry 15).

The ^19^F-NMR spectra contain two double doublets at −47 and −65 ppm with expected coupling constants for the axial and equatorial fluorine atoms: ^1^*J*_PFa_ = 915 Hz, ^1^*J*_PFe_ = 1025 Hz, and ^2^*J*_FaFe_ = 62 Hz. Apparently [2+2]-cycloaddition of the C=O group to the ylide **15** proceeds with high stereoselectivity, because the ^19^F- and ^31^P-NMR spectra show the signals belonging to the single diastereomer of the compounds **31**. The signals of the second diastereomer, the existence of which one can suppose as a consequence of the presence of two asymmetric endocyclic C-3 and C-4 carbon atoms are absent. The ^13^C-NMR spectra reveal the presence of the signals at 62.5 ppm (^1^*J*_CP_ = 150 Hz, ^2^*J*_CF_ = 53 Hz) and 75.6 ppm due to the C-3 and C-4 carbon atoms in complete accordance with the assigned structure of oxaphosphetane (Figure 1 and Figure 2).

**Scheme 20:**
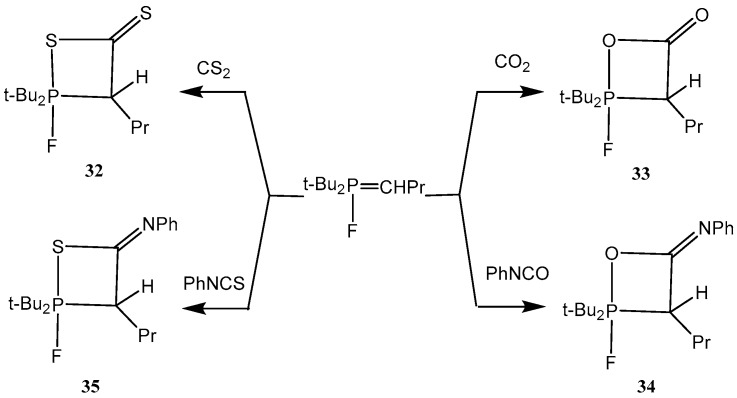
Addition of heterocumulenes (X=C=Y) to *P*-fluoroylides.

*P-*Fluoroylides add easily isocyanates, carbon dioxide and carbon disulfide with formation of oxaphosphetanes **32**–**35**. For example, the reaction of *P-*fluoroylides with phenyl isocyanate resulted in the formation of 2-fluorooxaphosphetane 34 which was stable during several hours at ambient temperature. Compound **34** was purified by crystallization in pentane and isolated as colorless crystalline matter. In IR spectra of compounds was found the strong band at 1730 cm^−1^ belonging to the C=O bond in a four-membered cycle. The ^31^Р-NMR spectra disclosed a doublet with constant ^1^*J*_PF_ = 780 Hz and a doublet with the same constant in ^19^F-NMR spectra. The oxaphosphetanes **32**–**35** convert at room temperature slowly and at heating quickly into phosphorylated heterocumulenes (ketenes, thioketenes, ketenimines, see Scheme 20) [29,37,38,39].

### 2.3. Properties of Oxaphosphetanes

**Scheme 21:**
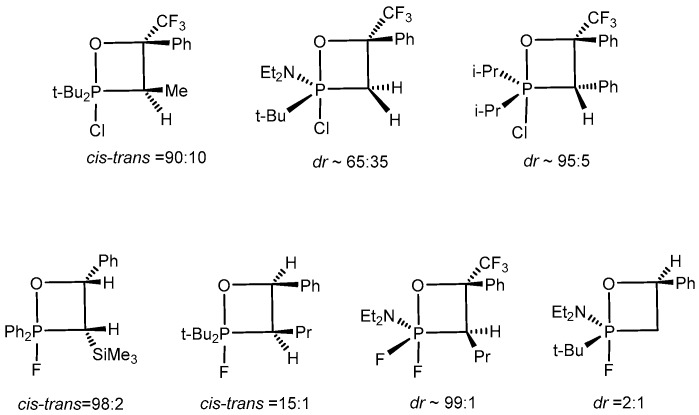
Diastereomeric composition of 2-chloro- and 2-fluoro-1,2λ^5^-oxaphosphetanes.

The cycloaddition of *P-*halogenylides to aldehydes and ketones proceeds stereoselectively and leads predominantly to the formation of one of the possible diastereomers of 2-halo-1,2λ^5^-oxaphosphetanes (Table 1 and Table 2). *P-*Haloylides (chloro- and bromo) react stereoselectively with aldehydes to give predominantly single diastereoisomers of 2-halo-oxaphosphetanes, bearing asymmetric endocyclic C-3 and C-4 carbon atoms (dr ~7:1–15:1). It was established by NMR that the ratio of diastereoisomers of 2-halooxaphosphetanes containing asymmetric atoms at C(3) and C(4) was within the limits ~99:1–90:10. The ratio of diastereomers after completion of the reaction was approximately 6:4–9:1. However on heating, as a result of permutational changes in the molecule, the ratio of diastereomers grew in favour of the thermodynamically more stable diastereomer. 2-Fluoro- and 2-chloroxaphosphetanes containing chiral phosphorus or endocyclic carbon atoms exist as mixtures of diastereomers whose ratio depends on the nature of the starting reagents (Scheme 21). The diastereomeric purity of the compounds, assessed by NMR spectroscopy, was 80%–96%. The reaction of α,α,α-trifluoroacetophenone with *P,P*-difluoroylides proceeded with very high stereo-selectivity to furnish only one diastereomer of compound. At the same time 2-halo-1,2λ^5^-oxa-phosphetanes bearing asymmetric phosphorus atom are formed with low stereoselectivity (dr~ 3:1).

**Scheme 22:**
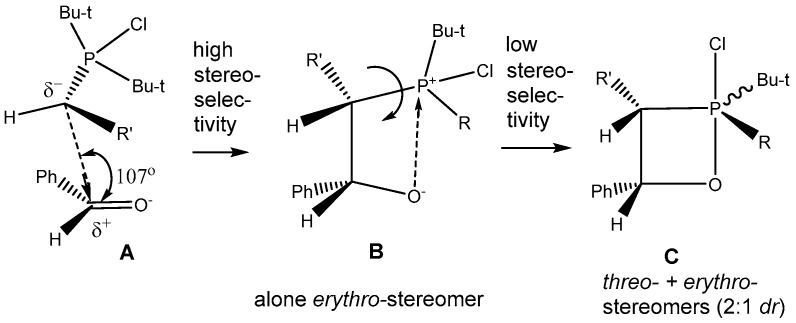
Mechanism of asynchronous [2+2]-сycloaddition of *P*-chloroylide to benzaldehyde.

**Figure 3:**
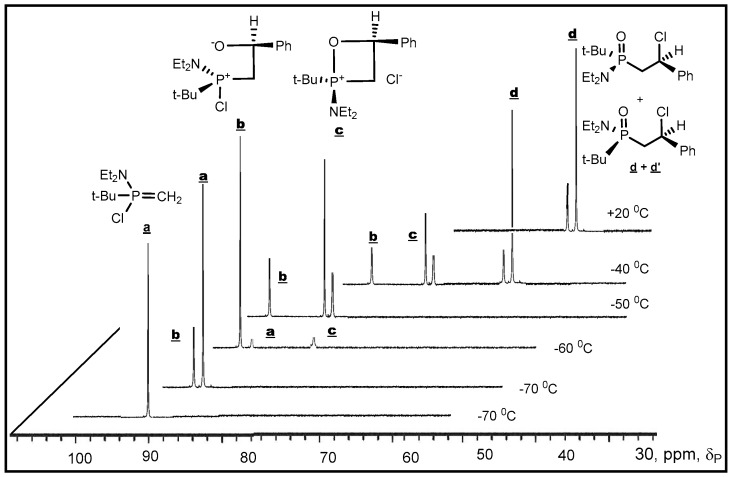
^31^P-(^1^Н-)NMR monitoring of the reaction of ylide **a** with PhCHO leading via the formation of betaine **b**, and oxaphosphetane **c** to the formation of β-chloroalkylphosphonates **d** and **d**′.

The reaction of *P-*halogenylides with aldehydes leads to the formation of an *erythro*-oxaphosphetanes with the oxygen in the apical position, because of the tendency of electron-acceptor atoms to occupy this position [40]. The preferred orientation of the transition state leading to the *erythro-*oxaphosphetane is stereoselective approach of the ylide nucleophilic center to the carbonyl group at an angle of 107°, with the double bonds of both reagents arranged in one plane. This was associated with the Burgi-Dunitz trajectory concept (Scheme 22) [41]. The first step of reaction stereoselectively leads to the formation of the betaine **B**. The conversion of reagents into oxaphosphetane requires minimum energy when it proceeds with the non-synchronous formation of bonds between the carbonyl and ylidic carbon atoms. On the second step the betaine converts into oxaphosphetane **C** with low stereoselectivity, because of free rotation around of the P-C bond [28,42]. This mechanism of asynchronous addition of *P-*haloylides to carbonyl compounds was confirmed by low temperature NMR investigations. The reaction of di-*tert*-butylсhlorphosphonium methylide with Ph(CF_3_)C=O was studied by ^31^Р-NMR at low temperature (−70–0 °C) in diethyl ether solution. At −70 °С was found only a decreasing signal of *P-*chloroylide (+114 ppm) and a growing signal of 2-chloro-1,2λ^5^-oxaphosphetane (+9.9 ppm). The signal which could be referred to betaine was not found, probably, because of the high speed of betaine cyclisation into 2-chloroxaphosphetane. However the reaction of *tert*-butyl(diethylamino)chlorophosphonium methylide with benzaldehyde began at −70 °С and led to the formation of *erythro-*betaine which was registered by increasing signal δ_P_ +106 ppm, located in more weaker field than the signal of initial *P-*chloroylide (+102 ppm) (Figure 3). The betaine formation was proceeded stereoselectively as in the ^31^P-(^1^Н-)NMR spectrum only one diastereomer signal was registered. The rise of temperature to −50 °С led to the formation of two signals of *threo* and *erythro*-oxaphosphetane diastereomers δ_Р_ + 92.6 and +84.6 ppm in the ratio of 2:1. Evidently, the formation of diastereomers proceeded via S*_N_*2@P substitution with inversion of configuration at the phosphorus atom [43]. The formation of isomers indicates on the nucleophilic substitution at asymmetric tetracoordinate phosphorus atom, proceeding with partial inversion of configuration. At −50–−40 °С the oxaphosphetane was converted into 2-chloralkylphosphine oxide, which was also formed as two *threo*- and *erythro*-diastereomers in the ratio of 2:1 (δ_Р_ +48.7 and +49.2 ppm). The conversion was completed at −20 °С. The *threo*- and *erythro* diastereomers were separated and isolated in pure state by chromatography and crystallization (m.p. 100 and 123 °С, see Table 3, entries 13, 14). Although at present many of the puzzling features about the reaction mechanism of ylides phosphorus with carbonyl compounds have been clarified, it seems there is no general mechanism, which could explain the progress of the reaction, transition states and stereochemistry. Nevertheless in case of *P-*chloroylide addition to aldehydes, on the basis of presented above stereochemical researches the asynchronous [2+2]-сycloaddition seems to be the most likely mechanism.

2-Halo-oxaphosphetanes exist in pentacoordinate form, but ionize under certain conditions with formation of tetracoordinate forms. In the mass spectra of 2-chlorooxaphosphetanes the peak of molecular ion was observed that indicated the covalent character of the P–Cl bond. All oxaphosphetanes show methyl carbons with ^l^*J*_PC_ values characteristic of equatorial placement in the trigonal bipyramid. The resonances of the methylene carbons of the phosphetane ring system lay downfield of the methyl carbons and the large measured ^l^*J*_PC_ values indicate equatorial placement. The four-membered ring is occupying an apical-equatorial plane where the O-P-C angle is 90°, and never a diequatorial plane or a diapical plane where the O-P-C angle would have to be 120 or 180°, respectively.

In the ^1^H-NMR spectra of the 2-halooxaphosphetanes possessing asymmetric carbon atom C^4^ the magnetic nonequivalence of protons СН^a^Н^b^ in the four-membered ring became apparent because the geminal spin-spin interaction arose between them. Each of the signals СН^a^ and СН^b^ was the double doublet with the coupling constant with phosphorus nucleus ^2^*J*_PH_ = 20–22 Hz and a constant of geminal interaction ^2^*J*_HH_ = 16–17 Hz. The chemical shifts ^31^P of 2-halogenoxaphosphetanes depended on the polarity of the solvent. Thus, in nonpolar solvents (diethyl ether, pentane) the signals of δ_P_ are located in the strong field of the ^31^P-NMR spectrum (from −3 to 10 ppm) that corresponds to the pentacoordinated state of the phosphorus atom. In polar solvents and especially in the presence of Lewis acids, like AlCl_3_, the values of δ_P_ were shifted downfield. For example, for 2-chloro-oxaphosphetanes the chemical shift of phosphorus, δ_P_ was as follows (ppm): 9 (pentane), 22 (СН_2_С1_2_), 30 (СНС1_3_), 35 (СН_3_СN), 45 (СНС1_3_ + А1С1_3_ (traces)). With the increase in amount of aluminum chloride up to equimolar level the value of δ_P_ 100 ppm was registered for 2-chlorooxaphosphetanes that was in accordance with the values of chemical shifts for the known alkoxyphosphonium salts and apparently indicated complete ionization of chlorophosphorane with the formation of phosphonium salt. The dependence of the chemical shift value of phosphorus in 2-chlorooxaphosphetanes on the solvent polarity and on the presence of Lewis acid is in accordance with the published data showing that chlorophosphoranes can be ionized with the formation of phosphonium structures. Herewith in the ^31^P-NMR spectrum the resultant signal is registered due to fast exchange in the phosphorus coordination **15A** 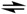 **15B**. The value of δ_P_ is shifted downfield proportionally to the increase in the phosphonium structure content, which in its turn depends on the solvent polarity. Substitution of electron withdrawing CF_3_ group at C4 atom by hydrogen atom destabilizes the oxaphosphetane cycle and considerably reinforces the ionization of Р-С1 bond. The stability of 2-halogenoxaphosphetanes decreases, and positive values δ_Р_ and ionization of Р-С1 bond increases in the sequence of substituents at С4 atom: СF_3_ > С_6_Н_4_F-4 >-C_6_N_5_ > С_6_Н_4_ОMе > H (Table 4) [25,30,31]. Chemical shifts of phosphorus in 2-bromo-1,2λ^5^-oxaphosphetanes (δ_Р_ = +20–+75 ppm) were in the weaker fields relatively to chemical shifts of chloro-oxaphosphetanes. Evidently high positive values δ_Р_ of 2-bromoxaphosphetanes is undoubtedly explained by bigger, than in case of 2-chlorooxaphosphetanes, contribution of phosphonium forms **15B** in the equilibrium 15**A** 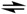 15**B**.

**Scheme 23:**
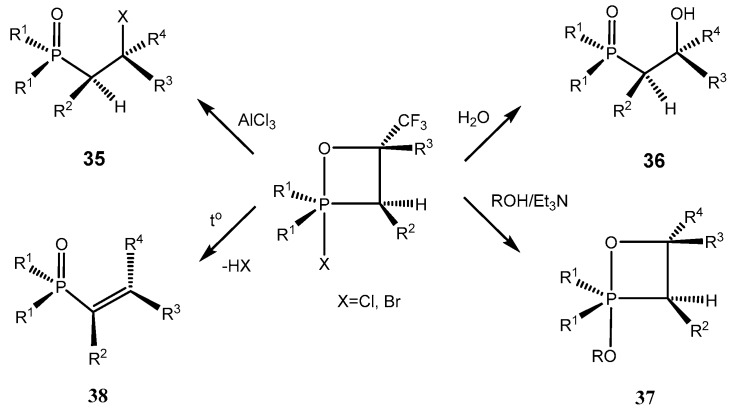
Chemical reactions of 2-chloro and 2-bromo-1,2λ^5^-oxaphosphetanes.

2-Chloro-1,2λ^5^-oxaphosphetanes enter into a number of interesting chemical conversions. Thus, 2- chloro-1,2λ^5^-oxaphosphetanes as a result of [1,3]-migration of chlorine atom to carbon atom underwent 2-chlorooxaphosphetane-2-chlorooxyphosphine oxide rearrangement to convert into the 2-chlorooxyphosphine oxides **35**. Thermal stability of 2-chloro-1,2λ^5^-oxaphosphetanes was decreased in case of oxaphosphetanes which not contain at С4 atom strong acceptor substituents, which rearranged into chloroalkylphosphonates at room temperature (Table 3). At heating 2-chloralkylphosphine oxides yield vinylphosphine oxides **38**. Hydrolysis of 2-chloro- and bromo-oxaphosphetanes led to the formation of 2-hydroxyalkylphosphine oxides **36** (Table 5)**.** The chlorine atom of 2-сhloro-1,2λ^5^-oxaphosphetanes is easily substituted on methoxy- or phenoxy groups by reaction with methanol or phenol in the presence of triethylamine with formation of 2-alkoxyoxaphosphetanes **37**, which at heating were converted into alkenes (Scheme 23, Table 1, entries 14, 15) [27,42].

**Scheme 24:**
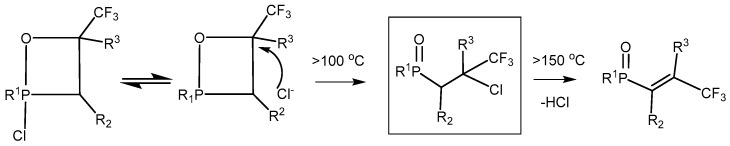
2-Chloro-1,2λ^5^-oxaphosphetane-2-chloroalkylphosphonate rearrangement.

The 2-chloro-1,2λ^5^-oxaphosphetanes not containing electron accepting groups at C-3 are unstable and rearrange easily into 2-cloroalkylphosphonates at temperature below +20 °C. The oxaphophetanes bearing at C4 electronegative CF_3_ groups convert into vinylphosphonates with elimination of hydrogen chloride at heading up to 150–160 °C. 2-Bromooxaphosphetanes are less stable than 2-chlorooxaphosphetanes and are converted into vinylphosphine oxides at room temperature. At heating (160–190 °С) 2-chloralkylphosphine oxides eliminate HCl to convert into vinylphosphine oxides (Scheme 24) [27,28,29,42].

**Scheme 25:**
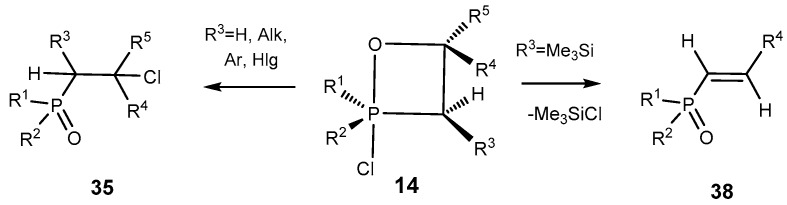
Conversions of 2-chloro-1,2λ^5^-oxaphosphetanes **14**.

The oxaphosphetanes bearing a trimethylsilyl group at C-3 atom eliminate the trimethylchlorosilane moiety and convert into the phosphorylated alkenes **38**. The conversion of oxaphosphetanes into alkenephosphonates proceeds at room temperature slowly, and at heating faster to give the alkenes **38** in good yields (Scheme 25). The phosphorylated alkenes **38** were purified by distillation under vacuum and isolated as pure compounds. The reaction of ylides **14** with aldehydes is regioselective. For example, the oxaphosphetanes obtained by reaction of silylated *P-*chloroylide **5** with terephthalic aldehyde depending on a ratio of initial reactants led to the formation of 1,4-bis-vinylphosphonobenzene or phosphonovinylbenzaldehyde, that represent interest as reactants for organic synthesis (See Table 6, entries 10, 11) [13,18,19,20].

**Scheme 26:**
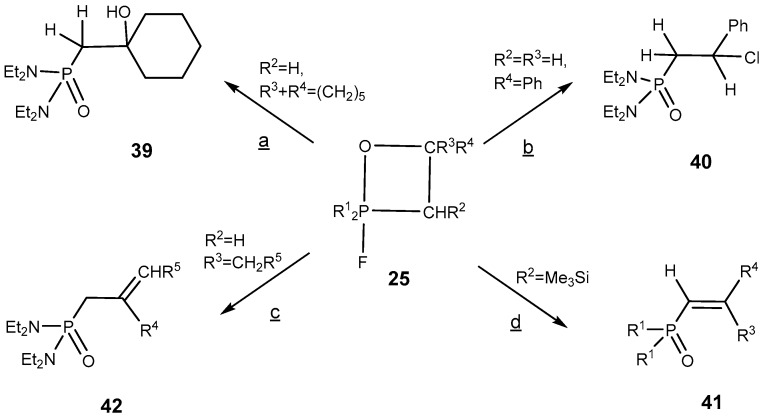
Chemical properties of 2-fluoro-1,2λ^5^-oxaphosphetanes. a = H_2_O (-HF); b = HCl, ether (-HF); c = 100–120 °C, ~0.5 h (-HF) or BF_3_·Et_2_O, +20 °C, 168 h; d = 60–80 °C (-Me_3_SiF).

The 2-fluoro-1,2λ^5^-oxaphosphetanes enter readily into a number of interesting organophosphorus compounds proceeding without Р—С bond cleavage [26]. Thus, the treatment of 2-fluorooxaphosphetanes **25** with ether solution of HCl led to the formation of 2-chloro-oxaphosphetanes **40**, which was isolated in good yield. At heating the 2-fluorooxaphosphetanes **25**, in contrast to triphenyloxaphosphetanes, convert into phosphorylated alkenes: vinylphosphonates or allylphosphonates. The direction of reaction depended on substituents R^2^ and R^3^ at C-3 and C-4 of oxaphosphetane cycle. The 2-fluorooxaphosphetanes bearing at C-3 R^2^ = H, Alk, Аr, and at C-4 R^3^ = Alkyl eliminated HF to convert into the allylphosphonates **42** [39,44,45]. The reaction was catalyzed by boron trifluoride etherate. At the same time the 2-fluorooxaphosphetanes **25** bearing at C-3 substituent R = Me_3_Si eliminated Me_3_SiF and afforded the vinylphosphonates **41** (Scheme 26, Table 7). This reaction represent a convenient method for the preparation of phosphorylated alkenes that are versatile building blocks for organic synthesis [45,46,47,48]. 2-Fluoro-1,2λ^5^-oxaphosphetanes containing two alkyl groups at C3 as well eliminated hydrogen fluoride and afforded the allylphosphonates **42** (Scheme 26, Table 8).

The reaction occurs by the 1,4-elimination as shown in Scheme 26 [25]. Opposite to 2,2,2-triphenyloxaphosphetanes, upon heating, the decyclization of 2-fluorooxaphosphetanes led to the formation of phosphorylated alkenes **41**, **42**.

**Scheme 27:**
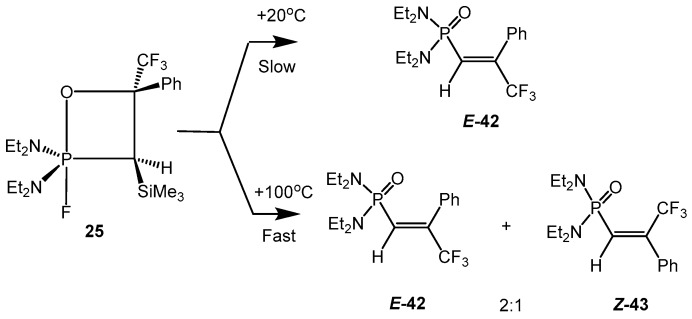
Effect of temperature on the stereoselectivity of 2-fluoro-1,2λ^5^-oxaphosphetane conversion into vinylphosphonates.

**Scheme 28:**
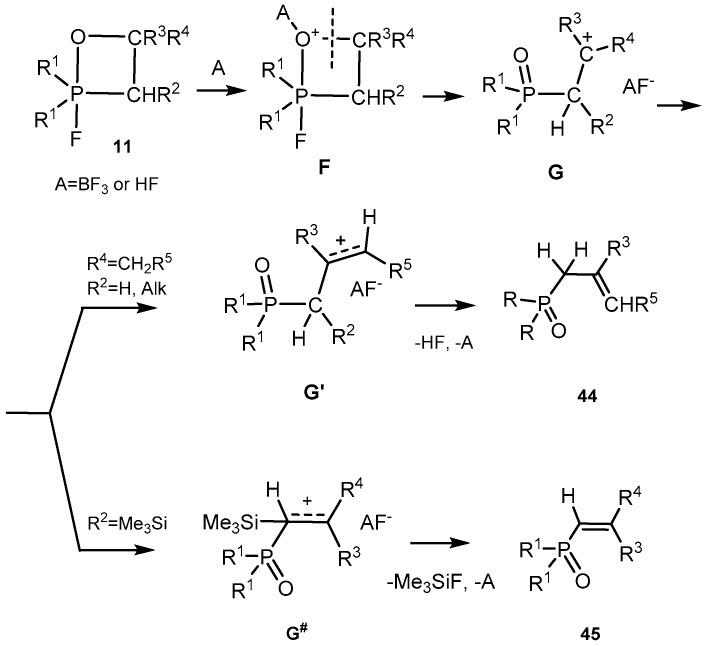
The mechanism for the 2-fluoro-1,2λ^5^-oxaphosphetane conversion into allyl- or vinylphosphonates **44**, **45**.

At heating to +100 °C the 2-fluoro-3-silyloxaphosphetanes **25**, bearing CF_3_ group at С-4, afforded a mixture of *E*- and *Z*-vinylphosphonates **29**, **30** in the ratio 2:1 with elimination of Me_3_SiF. However the slow conversion of oxaphosphetane at +20 °С during several days provided almost pure vinylphosphonates *E*-**29**, containing only 2%–3% of *Z*-isomer (Scheme 27) [26]. This effect was explained by formation of carbocation intermediate and rotation of substituents around the C-C bond (Scheme 28).

The conversion of 2-fluoro-1,2λ^5^-oxaphosphetanes bearing alkyl groups at C-3 into allylphosphonates **42** represents an interesting example of 1,4-elimination as Scheme 28 and Table 8 show [45]. The study of the reaction mechanism showed that Lewis and Broensted acids actively catalyze the conversion of 2-fluorooxaphosphetanes into allylphosphine oxides. The reaction is autocatalytic because the evolving hydrogen fluoride catalyzes the transition of 2-fluorooxaphosphetanes into allylphosphine oxides. The decomposition of protonated 2-fluorooxaphosphetanes leads to the formation of oxonium salts and carbocation intermediates under conditions of E*_N_*1 elimination. We suppose that the 2-fluorooxaphosphetanes under condition of acid catalysis (with HF or BF_3_) via the formation of an oxonium intermediate F, convert to carbocation intermediate **G** which has a planar configuration [46]. The removal of a proton from the carbocation intermediate **G** depends on electronic effects of the substituents. Alkyl groups possessing the +*I*-effect and the effect of hyperconjugation stabilize the positive charge and reduce the energy of intermediate **G** formation. Therefore, the intermediate **G′** leading to allylphosphonates is energetically more favorable than this one that is converted into vinylphosphonates.

In addition, even in case when the initial 2-fluorooxaphosphetanes **25** exist as a mixture of *threo-* and *erythro-*diastereomers, they converted into pure *E*-vinylphosphonates. Probably, this effect can be explained by rotation of substituents around the C-C bond in carbocation intermediate G. However, the presence of Me_3_Si at С-3 in **G**” leads to the elimination of Me_3_SiF, which has a high energy of formation, that creates a preference for the formation of vinylphosphonates **45** (Scheme 28) [26,40].

**Scheme 29:**
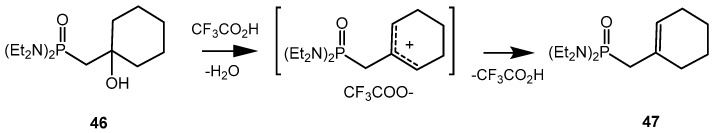
The conversion of hydroxyphosphonate **46** into the allylphosphonate **47**.

The formation of carbocation intermediate **G** was experimentally confirmed (Scheme 29). The treatment of 2-hydroxyphosphonate **46** with trifluoracetic acid and refluxing for several hours, generated the carbocation intermediate, as a result of acid-catalyzed dehydration of alcohol.

After that the carbocation intermediate is converted into allylphosphonate **47**, which is identical to the one obtained from 2-fluorooxaphosphetane **25** [26,40,45].

**Scheme 30:**
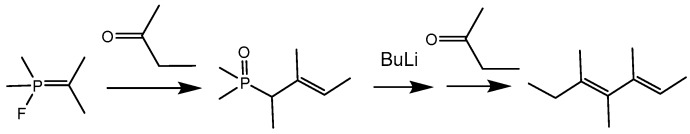
Synthetic application of allylphosphonates.

The reaction of *P-*fluoroylides with carbonyl compounds is a convenient method for the synthesis of allylphosphonates having various applications in the synthesis of naturally occurring compounds (Scheme 30) [45,46,47]. In this case, the carbonyl compounds can be used twice for the constructing of diene structures: first in reaction with *P-*fluoroylide and then in the Wittig reaction with allylphosphonate. This reaction was used for preparing analogs of the juvenile hormone.

**Scheme 31:**
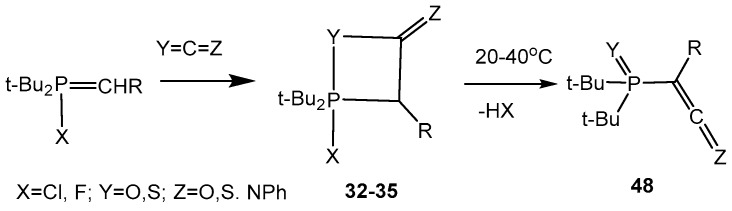
Preparation of phosphorylated ketenes, thioketenes, ketenimines **48** (Table 9).

The cycloadducts of *P-*fluoroylides with carbon dioxide or with carbon disulfide were isolated as colorless liquids or crystalline substances. Their structure was confirmed by the NMR spectra. Upon gentle heating or at room temperature these cycloadducts **32**–**35** (Table 2, entries 21, 22) are converted to phosphorylated ketenes or thioketenes **48** (Scheme 31). Stable [2+2]-cycloadducts of 2-fluoroylides with alkyl and aryl isothiocyanates were also synthesized and converted into phosphorylated ketenimines. The reaction of *P-*fluoroylides with carbon disulfide gives unstable cycloadducts that at temperatures above 0 °C converted completely into phosphorylated thioketenes in high yields. Phosphorylated thioketenes are red liquids distillable in vacuum and susceptible for various transformations [29,36,37]. The same phosphorylated ketenes or thioketenes **29** were prepared by reaction of *P-*cloroylides corresponding with CO_2_ and CS_2_ (See Scheme 31 and Table 9) [37,38,39].

## 3. Conclusions

In conclusion, this review has summarized the achievements in the synthesis and properties of stable four-membered phosphorus heterocycles-2-chloro-, 2-bromo- and 2-fluoro-1,2λ^5^-oxa-phosphetanes. These interesting compounds were obtained by reaction of *P-*halogenylides with various carbonyl compounds (aldehydes, ketones, isocyanates, carbon dioxide, and others). The 2-chloro and 2-bromo-1,2λ^5^-oxaphosphetanes, depending on the halogen nature and substituents at the α-carbon atom, underwent the rearrangement into 2-haloalkylphosphonates or with elimination of hydrogen halides were converted into *trans-*phosphorylated alkenes. Hydrolysis of 2-halo-1,2λ^5^-oxaphosphetanes led to the formation of 2-hydroxyalkylphosphonates. The 2-fluoro-oxaphosphetanes, bearing alkyl groups at the С-4 atom at heating as a result of 1,4-elimination of hydrogen fluoride are turned to allylphosphonates. Upon heating 3-silyl-2-fluoro-1,2λ^5^-oxaphosphetanes easily eliminate the trimethylsilylfluoride to convert into *E*-vinylphosphonates in high yields. These reactions provide efficient protocols for the preparation of various phosphorylated alkenes (vinylphosphonates, allylphosphonates, phosphorus ketenes, ketenimines, thioketenes) and can be useful for fine organic synthesis.

## Figures and Tables

**Table 1:** 2-Chloro- and 2-bromo-1,2λ^5^-oxaphosphetanes. 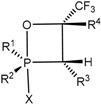

Entry	R^1^	R^2^	R^3^	R^4^	X	δ_P_	δ_F_	References
1	*t*-Bu	*t*-Bu	H	C_6_H_4_F-4	Cl	+ 9.0	+7.2 c (CF_3_); −32.9 (C_6_H_4_F)	[36,42]
2	*i*-Pr	*i*-Pr	Ph	Ph	Cl	+1.23; +0.53	+1.08; +1.64	[42]
3	*t*-Bu	*t*-Bu	H	Ph	Cl	+ 9.94	+1.0	[42]
4	*t*-Bu	*t*-Bu	H	An-4	Cl	+13.6	+7.35 c	[36,42]
5	*t*-Bu	*t*-Bu	H	CF_3_	Cl	+0.25	6.0	[36,42]
6	*t*-Bu	*t*-Bu	Me	Ph	Cl	+13.8; +11.65	+6.1	[42]
7	*t*-Bu	Et_2_N	H	Ph	Cl	+5.01; +9.13	+6.06; +6.09	[42]
8	*t*-Bu	*t*-Bu	Me	CF_3_	Cl	+2.53	+5.38;	[42]
9	*t*-Bu	Et_2_N	H	CF_3_	Cl	−2.6	+2.65 q; +2.94 q, ^1^*J*_FF_ 8	[42]
10	*t*-Bu	*t*-Bu	H	CF_3_	Br	+24.4	+6.25	[42]
11	*t*-Bu	*t*-Bu	H	Ph	Br	+46	+7.35	[42]
12	*t*-Bu	*t*-Bu	H	C_6_H_4_F-4	Br	+46	+5.6 (CF_3_); −34.2 (C_6_H_4_F)	[42]
13	*t*-Bu	*t*-Bu	H	An-4	Br	+60	+6.1	[42]
14	*t*-Bu	*t*-Bu	H	Ph	OMe	−14.6	+1.15	[42]
15	*t*-Bu	*t*-Bu	H	Ph	OPh	−12.8	+2.0	[42]

**Table 2:** 2-Fluoro-1,2λ^5^-oxaphosphetanes [26,32,37]. 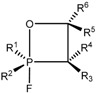

Entry	R^1^	R^2^	R^3^	R^4^	R^5^	R^6^	Yield, %	b.p., ^o^C (*р,* mmHg)	δ_P_, ppm.	δ_F_, ppm.	^1^*J*_PF,_ Hz
1	Et_2_N	Et_2_N	H	H	H	Bu	85	90 (0.06)	−42.15	31.30	767
2	Et_2_N	Et_2_N	H	H	H	C_6_H_13_	85	100 (0.06)	−42.25	31.12	766
3	Et_2_N	Et_2_N	H	H	Me	Me	70	75 (0.02)	−47.53	31.04	765
4	Et_2_N	Et_2_N	H	H	Me	Et	85	85 (0.06)	−49.20	31.04	765
5	Et_2_N	Et_2_N	H	H	Me	Ph	99	^a^	−44.00	32.00	766
6	Et_2_N	Et_2_N	H	H	(CH_2_)_5_		90	110 (0.06)	−44.60	34.90	766
7	Et_2_N	Et_2_N	H	Me	(CH_2_)_5_		99	^a^	−44.00	21.00	853
8	Et_2_N	Et_2_N	H	H	Ph	Ph	99	^a^	−41.60	34.90	766
9	Et_2_N	Et_2_N	H	H	H	Ph	99	^a^	−42.13	31.18	768
10	Et_2_N	Et_2_N	Cl	Cl	H	Pr	90	^a^	−47.00	5.40	853
11	Et_2_N	Et_2_N	Br	Br	H	Pr		^a^	−56.03	5.80	842
12	Et_2_N	Et_2_N	H	Me	CF_3_	Ph	80	120 (0.06)	−36.39; −38.27 (7:1) ^b^	−0.4	795; 795
13	Ph	Ph	Ph	H	CH_3_	H	95	^a^	−43.50	47.8	670
14	Et_2_N	F	H	Pr	CF_3_	Ph	85	105 (0.06)	−26.7 (99% dr)	−46.9, −65 (CF_3_)	915; 1025
15	Et_2_N	F	H	Pr	CF_3_	4-Tol	80	108 (0.06)	−26.5 (99% dr)	−46.6, −65 (CF_3_)	915; 1025
16	Et_2_N	Et_2_N	H	Ph	CF_3_	Ph	85	<20 ^c^	–38.96	27	792.6
17	*t*-Bu	*t*-Bu	H	Pr	CF_3_	Ph	70	125 (0.06)	−7.73, −8.79 (9:1) ^b^	−1.03	768; 770
18	*t*-Bu	*t*-Bu	H	Pr	H	Ph	75	120 (0.08)	−10.1; −9.62 (15:1) ^b^	9.9	762; 762
19	*t*-Bu	*t*-Bu	H	H	H	Ph	70	<20 ^c^	−5.9	13	750
20	Et_2_N	Et_2_N	H	*i*-Pr	H	Ph	70	110 (0.06)	−33.7; −33.54 (15:1) ^b^	7.2	827; 827
21	*t*-Bu	*t*-Bu	H	Pr	C=NPh		50	84–86 ^c^	−18.85	5.55	820
22	*t*-Bu	*t*-Bu	H	Pr	C=O			^a^	20.01	−26.23	785
23	Ph	Ph	Ph	SiMe_3_	Ph	H	85	^a^	−43.5	28.76	670
24	Et_2_N	Et_2_N	H	SiMe_3_	Ph	H	90	^a^	−39.18; −38.83 (3:2) ^b^	−40.19; −8.84	670
25	*t*-Bu	*t*-Bu	H	SiMe_3_	Ph	H	90	^a^	−11.0	-	768
26	Et_2_N	Et_2_N	H	SiMe_3_	Pr	H	90	^a^	−39.27; −42.11 (4:1) ^b^	−43.71; −42.61	768
27	Et_2_N	Et_2_N	H	SiMe_3_	Bu	H	95	^a^	−39.22; −42.32 (4:1) ^b^	−43.71; −42.61	768
28	Et_2_N	Et_2_N	H	SiMe_3_	C_6_H_13_	H	90	^a^	−39.20; −42.10 (3:1) ^b^	−43.71; −42.61	763
29	Et_2_N		H	SiMe_3_	C_8_H_17_	H	90	^a^	−39.88; 42.20 (4:1) ^b^	−42.85; −42.07	763
30	Et-N		H	SiMe_3_	CF_3_	Ph	95	^a^	−38.60; −37.45 (3:1) ^b^	−41.11 −49.01	777

^a^ Oil; ^b^ Diastereomers; ^c^ melting point (m.p.).

**Table 3:** 2-Chloroalkylphosphine oxides **35** (Scheme 23) [36,42]. 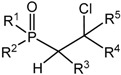

N	R^1^	R^2^	R^3^′	R^4^	R^5^	Yield, %	m.p./b.p. °C	δ_P_
1	*t*-Bu	*t*-Bu	H	H	CF_3_	60	62 (pentane)	57.8
2	*t*-Bu	*t*-Bu	H	Me	CF_3_	60	86 (hexane)	57.0
3	*t*-Bu	*t*-Bu	H	Pr	CF_3_	60	88 (hexane)	57.0
4	*t*-Bu	*t*-Bu	H	Ph	CF_3_	75	112.5 (benzene)	57.2
5	*t*-Bu	*t*-Bu	H	C_6_H_4_NO_2_	CF_3_	70	139 (hexane)	59
6	*t*-Bu	*t*-Bu	Me	C_6_H_4_NO_2_	CF_3_	70	131 (hexane)	60.1
7	*t*-Bu	*t*-Bu	Pr	C_6_H_4_NO_2_	CF_3_	60	143.5 (hexane)	60.0
8	Me_2_N	Me_2_N	H	Pr	CF_3_	60	b.p. 95 (0.06 mmHg)	45.8
9	Et_2_N	Et_2_N	H	Ph	CF_3_	40	123 (ether-pentane)	46.0
10	Et_2_N	Et_2_N	H	Ph	CF_3_	20	160 (hexane)	46.3
11	*i*-Pr_2_N	*i*-Pr_2_N	Me	Ph	H	50	107–111	34.4
12	*t*-Bu	Ph	H	Ph	H	75	oil	57.9; 58.3 (10:1)
13	*t*-Bu	Et_2_N	H	H	Ph	20	100 (hexane)	46.3
14	*t*-Bu	Et_2_N	H	Ph	H	40	123 (ether-pentane)	46.0

**Table 4:** Effect of solvent on δ_P_ of 2-chloro and 2-bromo-1,2λ^5^-oxaphosphetanes. 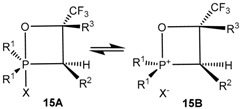

R^3^	СF_3_	С_6_Н_4_F	С_6_Н_5_	С_6_Н_4_ОМе
δ_P_ (СНСl_3_), Нlg = С1	+0.25	+9.0	+10.0	+13.6
δ_P_ (ether), Нlg = Вг	+25.0	+60.0	+65.0	+75.0

**Table 5:** 2-Hydroxyalkylphosphine oxides **23** (Scheme 23) [39,42]. 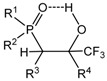

Entry	R^1^	R^2^	R^3^	R^4^	Yield, %	m.p., °C	δ_Р_	δ_F_
1	*i*-Pr	*i*-Pr	Ph	Ph	90	131–132	+66.0	+5.63
2	*t*-Bu	*t*-Bu	H	Ph	90	155–156	+68.25	−1.78
3	*t*-Bu	*t*-Bu	H	C_6_H_4_F-*p*	85	132	+68.7	−2.02; −34.5
4	*t*-Bu	*t*-Bu	H	An-*p*	70	140–141	+68.5	+4.12
5	*t*-Bu	*t*-Bu	Me	Ph	50	152	+73.4	+3.13
6	*t*-Bu	Et_2_N	Me	CF_3_	50	108	+53.4	+8.1
7	*t*-Bu	Et_2_N	H	Ph	65	150	+57.4	−3.06
8	*t*-Bu	Et_2_N	H	CF_3_	90	46–48	+56.6	+6.1
9	*t*-Bu	*t*-Bu	H	CF_3_	90	88-89	+67.5	+6.25

**Table 6:** Vinylphophonates **38** (Scheme 25). 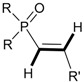

Entry	R	R′	Reaction Conditions			Yields, %	References
			Time, h	t °C	Solvent		
1	*t*-Bu	Ph	0.5	150	a	80	[25,32]
2	EtO	Ph	2	25–100	ether	80	[25,32]
3	EtO	2-BrC_6_H_4_	6–8	25–100	ether	60	[25,32]
4	*i*-PrO	Ph	2	25–100	ether	60	[25,32]
5	Et_2_N	Ph	14	25	ether	80	[25,32]
6	Et_2_N	2-FC_6_H_4_	24	25	ether	60	[25,32]
8	Et_2_N	CH=CHPh	18	25	ether	80	[25,32]
9	Et_2_N	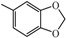	24	25	THF	70	[25,32]
9	EtO	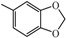	48	35	THF	65	[25,32]
10	Et_2_N	C_6_H_4_CHO-*p*	25	18	THF	50	[25,32]
11	Et_2_N	R_2_P(O)CH=CHC_6_H_4_	25	24	THF	50	[25,32]
12	*i*-PrO	R_2_P(O)CH=CHC_6_H_4_	25	24	THF	50	[25,32]

a without solvent.

**Table 7:** Vinylphosphonates **41** (Scheme 26) [26]. 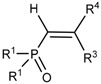

Entry	R^1^	R^3^	R^4^	Yield, % ^a^	b.p. °C (*p* mmHg)/m.p. °C (Solvent)	δ_P_, ppm
1	*t*-Bu	H	Ph	50	138 (heptane)	35.0
2	Ph	H	Ph	40	165 (heptane)	22.0
3	Et_2_N	H	Ph	85	103.5 (hexane)	24.7
4	Et_2_N	H	Me	80	120 (0.05)	24.7
5	Et_2_N	H	Pr	72	120 (0.05)	24.3
6	Et_2_N	H	Bu	68	120–123 (0.04)	23.95
7	Et_2_N	H	C_6_H_13_	80	135 (0.06)	24.1
8	Et_2_N	H	C_8_H_17_	79	145–150 (0.05)	24.1
9	Et_2_N	H	PhCH=CH-	60	170 (0.06)	23.30
10	Et_2_N	H	Me_2_C=CHCH_2_CH_2_C(Me)CH=C-	35	160 (0.08)	25.0
11	Et_2_N	CF_3_	Ph	74	140 (0.03)	17.0; 18.7 ^b^
12	Et_2_N	H	-C_6_H_4_CHO-4	50	98 (hexane)	23.7
13	Et_2_N	H	-C_6_H_4_CH=CHP(O)(NEt_2_)_2_	50	190 (heptane)	24.8

^a^ yield of the isolated product; ^b^ mixture of *E/Z*-diastereomers in 97:3 ratio.

**Table 8:** Allylphosphonates **28**. 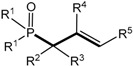

Entry	R^1^	R^2^	R^3^	R^4^	R^5^	Yield, %	b.p. °С (*р*, mmHg)	References
1	Et_2_N	H	H	H	Pr	70	120 (0.06)	[40,45]
2	Et_2_N	H	H	H	C_5_H_11_	70	140 (0.08)	[40,45]
3	Et_2_N	H	H	Me	H	55	105 (0.08)	[40,45]
4	Et_2_N	H	H	Me	Me	60	95 (0.06)	[40,43]
5	Et_2_N	H	H	Ph	H	65	145 (0.08)	[40]
6	Et_2_N	H	H	(CH_2_)_4_		85	145 (0.08)	[26,45]
7	Et_2_N	H	Me	(CH_2_)_4_		75	110 (0.06)	[40,45]
8	Et_2_N	Me	Me	(CH_2_)_4_		70	110 (0.06)	[40,45]

**Table 9:** Preparation of phosphorylated ketenes, thioketenes, ketenimines **48** (Scheme 31). 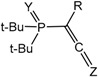

Entry	R	X	Y	Z	Yield, %	b.p. °C (mmHg)	References
1	H	Cl	O	O	80	a	[36,39]
2	Me	F	O	O	70	98 (0.06)	[37]
3	Me	Cl	O	O	85	98 (0.06)	[36,39]
4	Pr	F	O	O	60	102 (0.06)	[37]
5	Pr	Cl	O	O	69	102 (0.06)	[36,39]
6	Me	F	O	NPh	45	150 (0.06)	[37]
7	Pr	F	O	NPh	45	150 (0.06)	[37]
8	Me	Cl	S	S	75	95 (0.06)	[38]
9	Pr	Cl	S	S	80	115 (0.06)	[38]
10	Pr	F	S	S	70	115–120 (0.06)	[37,38]
11	*i-*Pr	Cl	S	S	80	105 (0.06)	[38]

a unstable compound.
